# Recent advances in deep brain stimulation in psychiatric disorders

**DOI:** 10.12688/f1000research.14187.1

**Published:** 2018-06-05

**Authors:** Anne-Hélène Clair, William Haynes, Luc Mallet

**Affiliations:** 1 Sorbonne University, UPMC Paris 06 University, INSERM, CNRS, Institut du Cerveau et de la Moelle épinière, Paris, France; 2 Neurosurgery department, University Hospital of Montpellier, Montpellier, France; 3 Psychiatry and Addictology Department – Neurosurgery Department, Personalized Neurology & Psychiatry University Department, University Hospitals Henri Mondor – Albert Chenevier, Créteil, France

**Keywords:** deep brain stimulation, obsessive-compulsive disorder, major depression, gilles de la Tourette syndrome, psychosurgery

## Abstract

Deep brain stimulation (DBS) has been offered to patients suffering of severe and resistant neuropsychiatric disorders like Obsessive Compulsive Disorder (OCD), Gilles de la Tourette Syndrome (TS) and Major Depression (MDD). Modulation of several targets within the cortico-striato-thalamo-cortical circuits can lead to a decrease of symptom severity in those patients. This review focuses on the recent clinical outcomes in DBS in psychiatric disorders. Studies on OCD and TS are now focusing on the long-term effects of DBS, with encouraging results regarding not only the decrease of symptoms, but also quality of life. They also highlighted efficient adjuvant techniques, like cognitive and behavioural therapy and support programs, to enhance an often-partial response to DBS. The application of DBS for MDD is more recent and, despite encouraging initial open-label studies, two large randomised studies have failed to demonstrate an efficacy of DBS in MDD according to evidence-based medicine criteria. Last years, DBS was also tested in other resistant psychiatric disorders, as anorexia nervosa and addiction, with encouraging preliminary results. However, today, no target – whatever the disease – can meet the criteria for clinical efficacy as recently defined by an international committee for neurosurgery for psychiatric disorders. Consequently, DBS in psychiatric disorders still needs to proceed within the frame of clinical trials.

## Introduction

Deep brain stimulation (DBS) is a neurosurgical technique based on the stereotactic implantation of quadripolar electrodes delivering high-frequency current in a specific subcortical or deep cortical structure. It is usually indicated to decrease symptoms in neurological disorders, such as the hypodopaminergic symptom spectrum associated with Parkinson’s disease (PD). The reversibility of DBS, supporting its ethically “acceptable” status, combined with the improvement of psychiatric symptoms after lesional surgeries has certainly led to the transition towards its application in psychiatric disorders ^1^. Patients with severe debilitating disease, resistant to medical and psychotherapeutic optimal treatment, were enrolled in studies modulating cortico-striato-thalamo-cortical circuits with DBS.

DBS was first used to improve obsessive-compulsive disorder (OCD) ^2^ and Gilles de la Tourette syndrome (TS) ^3^ symptoms in 1999. In both disorders, patients feel compelled to perform repetitive motor and vocal tics (in TS) or repetitive behaviour associated with intrusive ideas (compulsions and obsessions) (in OCD). Based on the efficacy of previous lesional surgeries ^1^, the first targets were the anterior limb of internal capsule (ALIC) ^2^ in OCD and the centromedian-parafascicular complex (CM-Pf) in TS ^3^; 3 years later, an important decrease of comorbid OCD symptoms was reported in two parkinsonian patients stimulated in the subthalamic nucleus (STN) ^4^. The same year, given the effects of globus pallidus internus (GPi) DBS on hyperkinesia in PD, a patient with TS was stimulated in the postero-ventral GPi and showed postoperative clinical improvement ^5^. Then, based on pathophysiological evidence involving the subgenual cortex (BA 25, also termed Cg25) in major depressive disorder (MDD), the modulation of this structure with DBS was tested and led to an improvement of MDD symptoms ^6^. Here, we will present the recent developments of DBS in psychiatry, including the clinical outcomes for OCD, TS, and MDD patients and other emerging psychiatric applications.

## Obsessive-compulsive disorder

Randomised controlled studies have confirmed the initial reports by B. Nuttin ^2^ and L. Mallet ^4^ and highlighted two main targets for OCD: the striatal region (including the ALIC, the nucleus accumbens, the ventral caudate nucleus, and the ventral capsule/ventral striatum) ^7– 9^ and the STN ^10^. Two recent meta-analyses found that DBS leads to a global decrease of about 45% of OCD symptom severity with 60% of patients considered responders ^11, 12^.

Recent studies are now focusing on the long-term effects of DBS, which represents a decisive step to consider a broader application of DBS for OCD. In agreement with the report from Greenberg *et al*. 3 years after DBS in the striatal region ^7^, a stability of the initial decrease of OCD symptoms has been reported until 9 years after DBS ^13, 14^. Case reports that also suggest a stability of symptom decrease 6 months ^15^ and 10 years after STN stimulation ^16, 17^ have been published.

In addition, one may wonder whether the evaluation of DBS efficacy is limited to the decrease of OCD symptoms (as measured by the Yale-Brown Obsessive Compulsive Scale, or Y-BOCS). The recent focus on quality of life is related to a chronological (that is, after proving symptoms reduction), historical (that is, regarding the past misconducts in psychosurgery), and transnosographic (see Agid *et al*. ^18^) legacy. Indeed, the team of D. Denys has chosen to follow its initial cohort ^9^ by evaluating as a primary outcome the quality of life and subjective lived experience of the patients stimulated in the striatal region ^13, 19, 20^. After 3 to 5 years of DBS, patients reported a significant improvement in quality of life ^13^. Curiously, it improved in responders and non-responders to DBS as defined by Y-BOCS fluctuations, thereby questioning the psychometric limits and the relevance of this primary outcome measure for severe populations. In addition to the previously known effects of striatal DBS (for example, mood and anxiety), patients of this cohort reported that they became “more easy and loose”, “more self-confident”, and “expressive” but also experienced “difficulties in their relationship with partner” after DBS ^19^. Changes regarding the perception of themselves, their families, and other problems with social adjustments have been noticed in parkinsonian patients after clinical improvement by STN DBS ^21^. Thus, the psychological and social changes experienced by patients with OCD could be unspecific and highlight the lack of control groups in the studies on DBS for OCD ^13, 19^. Currently, a European multicentre study is comparing the quality of life of severe OCD patients stimulated in the STN versus optimised conventional treatment ^17^.

In the study by De Haan *et al*., we also noticed that patients reported an effect of DBS on the ability to engage in cognitive behavioural therapy (CBT) ^19^. Notwithstanding that almost all of the OCD patients included in DBS studies had initially failed to complete CBT, the cohort of D. Denys *et al*. ^9^ experienced an additional decrease of OCD symptoms after post-operative CBT ^22^. This interesting result places CBT as a potential strategy to enhance an often-partial response to striatal DBS in OCD and raises the interest in assessing this effect after STN stimulation.

Finally, the search for an optimal target in OCD is ongoing. Although a recent meta-analysis failed to find a significant difference between the efficacy of DBS in the striatal region and STN ^12^, a multicentre randomised trial is currently comparing the efficacy and safety of ventral capsule/ventral striatum versus STN DBS for OCD (ClinicalTrials.gov identifier NCT01329133). Otherwise, a case study reported a decrease in OCD symptoms after DBS of the medial forebrain bundle (MFB) ^23^, a promising target in MDD ^24^.

## Gilles de la Tourette syndrome

In the same way as OCD, the promising results reported in the two initial case studies ^3, 5^ were replicated, identifying two main targets for TS: the thalamus (including CM-Pf, dorsomedial nucleus, ventro-anterior and ventro-lateral) and the GPi (including the motor part [that is, postero-ventral] and associative-limbic part [that is, antero-medial]). For both targets, a recent meta-analysis also confirmed the efficacy of DBS with a median improvement of 52% in tic severity ^25^. However, this encouraging result is based mostly on open-label studies. It included only three randomised controlled studies, which had contradictory results, obtained from small samples, and most of them targeting the thalamus ^26– 28^. Thus, the efficacy of DBS for TS seems to be lacking evidence, especially in the GPi, a promising target with fewer side effects than the thalamus.

With the aim of providing evidence of the efficacy of GPi DBS on TS, two double-blind cross-over studies were recently conducted in larger samples ^29, 30^. Unfortunately, although the team of Kefalopoulou ^29^ found a significant decrease of (only) 15% in tic severity during the double-blind phase, no significant difference was measured by Welter *et al*. ^30^. However, for both, the improvement went up to 40% during the (open-label) follow-up period, and there were many inter-individual differences. This clearly highlights the difficulties in finding results in double-blind studies that are as encouraging as those in open-label ones. The placebo effect but also methodological issues (for example, duration of the blind period and limitation of the stimulation settings to preserve masking) and the chosen target (motor or associative-limbic part of the GPi) could explain this difference ^29, 30^. These limitations would probably be examined by the innovative database ^31^ recently created to consider new guidelines for trials using DBS in TS. The aim of this database was also to share and gather clinical experiences with DBS in TS ^31^, which offer a faster accumulation of evidence for DBS efficacy.

Recent studies are now interested in the long-term effects of DBS, as in OCD. Long-term side effects already reported after thalamic stimulation ^32^ have even led some authors to propose a different target to their patients with TS ^33^. Conversely, the long-term follow-up of TS patients stimulated in the GPi gave encouraging results ^34, 35^, including an improvement in quality of life ^36^. Unfortunately, as already reported in patients with OCD ^19, 20^ and PD ^21^, patients have difficulties in coping with changes after DBS ^36^. Consistently, the author stressed the importance of a multidisciplinary team and the need to approach patients about “how life will continue with or *without* tics” ^36^. This relevant consideration has already been successfully tested in PD patients with a perioperative psychoeducation program, leading to better social adaptation and a decrease of anxiety and depression ^37^. Given the numerous psychiatric diseases often comorbid to TS, such a support program could be an interesting strategy to increase an often-partial response to DBS in TS.

## Major depressive disorder

Although the literature on DBS for patients with MDD is relatively recent, numerous open-label studies have highlighted a decrease of depressive symptoms after DBS. Indeed, the results of the first trial stimulating Cg25 ^6^ were confirmed by subsequent studies ^38– 40^. As is the case for OCD, DBS of the ventral striatal region leads to improvement of depressive symptoms in open-label studies ^41– 43^.

Recently, the efficacy of DBS in MDD was tested in two large double-blind randomised sponsored studies. Inclusions of patients in these studies began shortly after the initial publication by Mayberg *et al*. ^6^, probably motivated by the influence of high-level studies on a wider application of DBS and the frequency of MDD. Targeting the striatal region, a first study planned to include more than 200 MDD patients for DBS ^44^. It was prematurely interrupted after including (only) 29 patients, as no significant difference was measured between the stimulated and non-stimulated groups. The same was observed for the largest DBS study in psychiatry, which failed to find significant differences after the stimulation of Cg25 among the 90 MDD patients included ^45^. These disappointing results are counterbalanced by the recent demonstration of significant improvement in MDD symptoms after ALIC DBS (with 40% of responders) in a third trial ^46^. This last study is still the only randomised controlled trial to show a significant effect of DBS on depression.

Taken together, these recent results highlighted the safety of DBS in MDD. However, one cannot conclude whether DBS has an effect on depression. Trying to find the reasons for this “failure” is a current debate within the DBS community. Some have pointed to the methodology of the randomised trials, specifically the way (for example, the delay and the algorithm used) to find the optimal stimulation parameters, and even the design of the study (for example, a cross-over versus parallel design and the duration between inclusion and blind period). Furthermore, the clinical characteristics of the patients with MDD are criticised, especially the duration of symptoms in some patients (up to 12 years) that could lead to a potential delayed effect of DBS ^45^ or be a measured manifestation of a special clinical phenotype of non-responder patients. The fact that the results have not been very encouraging until now reasonably raises the question of whether or not to continue trials with this invasive procedure in psychiatric patients.

However, these negative results could be explained by a target issue. Indeed, a dramatic improvement of depressive symptoms was measured after DBS of the supero-lateral medial forebrain bundle (slMFB) in open-label studies ^24, 47^. Now, one could wait for the results from a randomised study targeting this structure in MDD ^48^. They would be of particular interest to confirm, as 75% of patients are still responders 1 year after the start of DBS and have no serious side effects ^48^.

## Other psychiatric disorders

In recent years, several successful cases of DBS application to different neuropsychiatric disorders have been reported. As mentioned by the authors, those cases are in fact indicative of forthcoming open-label studies. If all of them succeed, we will know whether DBS of the slMFB decreases depressive symptoms in bipolar patients ^49^, whether the stimulation of baso-lateral amygdala improves resistant post-traumatic stress disorder ^50^, and whether DBS of the striatal region is effective in heroin ^51^ or cocaine ^52^ addiction or even in schizophrenia ^53^.

In patients with resistant anorexia nervosa (AN), encouraging first results were obtained on their body mass index (BMI) after the stimulation of Cg25 ^54^. Even if the first open-label study, including six AN patients stimulated in Cg25, led to disappointing results concerning the evolution of BMI ^55^, a significant increase of BMI was then measured in a larger group (16 patients with AN) stimulated in the same target ^54^. Additionally to an increase in BMI, patients have experienced an improvement in mood and anxiety. As the same team have already used this target to decrease depressive symptoms in MDD ^6, 38^, one cannot exclude a positive bias of mood on the primary outcome measure (BMI). Other open-label trials are ongoing to replicate these results and to test the efficacy of other targets (especially the striatal region) in AN (ClinicalTrials.gov identifiers NCT03168893 and NCT01924598).

## Conclusions

Long-term effects of DBS are encouraging about both the absence of relapse and the improvement in quality of life in severe and resistant TS and OCD. On the other hand, recent years have been marked by disappointing results of randomised studies in DBS for TS and MDD.

This raises the question of DBS’ efficacy for psychiatric disorders, framed by a growing concern for ethics ^56, 57^. Key principles of medical ethics are discussed, such as the ability to make an autonomous decision (also suggesting giving informed consent) or authorize a research procedure ^58^ or changes in personality following DBS ^20^. A consensus document on ethical and scientific conduct for psychiatric surgery was recently published ^56^. This document also mentioned that to make a conclusion regarding DBS’ efficiency (for a specific psychiatric population stimulated in a specific target), at least two studies managed by two different teams are necessary ^56^. Today, despite numerous randomised trials, no target in any psychiatric disease can meet these criteria.

This could be partially explained by heterogeneous clinical manifestations of psychiatric patients within a common diagnosis. Indeed, the DBS of a defined target may modulate the symptoms linked to a specific dysfunctional and partially overlapping neuronal circuit involved in psychiatric manifestations ^59^.

Shared databases, as recently initiated for DBS in TS, are an encouraging way to promote scientific reflexion and hopefully improve clinical efficacy of DBS in TS and maybe in other psychiatric disorders. This could also contribute to the identification of optimal targets for specific subgroups of patients, paving the way to precision medicine in psychiatry for severe and refractory mental disorders.
